# Quantitative blood flow measurement in rat brain with multiphase arterial spin labelling magnetic resonance imaging

**DOI:** 10.1177/0271678X18756218

**Published:** 2018-03-02

**Authors:** James R Larkin, Manon A Simard, Alexandre A Khrapitchev, James A Meakin, Thomas W Okell, Martin Craig, Kevin J Ray, Peter Jezzard, Michael A Chappell, Nicola R Sibson

**Affiliations:** 1 Department of Oncology, Cancer Research UK & Medical Research Council Oxford Institute for Radiation Oncology, University of Oxford, Oxford, UK; 2 Wellcome Centre for Integrative Neuroimaging, FMRIB Division, University of Oxford, John Radcliffe Hospital, Headington, Oxford, UK; 3 Institute of Biomedical Engineering, University of Oxford, Oxford, UK

**Keywords:** Arterial spin labelling, autoradiography, cerebral blood flow, multiphase, rats

## Abstract

Cerebral blood flow is an important parameter in many diseases and functional studies that can be accurately measured in humans using arterial spin labelling (ASL) MRI. However, although rat models are frequently used for preclinical studies of both human disease and brain function, rat CBF measurements show poor consistency between studies. This lack of reproducibility is due, partly, to the smaller size and differing head geometry of rats compared to humans, as well as the differing analysis methodologies employed and higher field strengths used for preclinical MRI. To address these issues, we have implemented, optimised and validated a multiphase pseudo-continuous ASL technique, which overcomes many of the limitations of rat CBF measurement. Three rat strains (Wistar, Sprague Dawley and Berlin Druckrey IX) were used, and CBF values validated against gold-standard autoradiography measurements. Label positioning was found to be optimal at 45°, while post-label delay was optimised to 0.55 s. Whole brain CBF measures were 109 ± 22, 111 ± 18 and 100 ± 15 mL/100 g/min by multiphase pCASL, and 108 ± 12, 116 ± 14 and 122 ± 16 mL/100 g/min by autoradiography in Wistar, SD and BDIX cohorts, respectively. Tumour model analysis shows that the developed methods also apply in disease states. Thus, optimised multiphase pCASL provides robust, reproducible and non-invasive measurement of CBF in rats.

## Introduction

Arterial spin labelling (ASL) is increasingly gaining popularity in the clinic for its functional MRI (fMRI) capabilities, as well as for perfusion analysis in the brain in diseases such as stroke^1,2^ and cancer.^3,4^ Despite its apparent simplicity, the many variations of ASL have complicated its implementation in routine clinical practice. To this end, the ASL ‘white paper’ was published in 2015 and gave recommended clinical guidelines for the purpose of standardising ASL across multiple scanners and centres.^5^ The final recommendations of the white paper include using a pseudo-continuous ASL (pCASL) labelling approach combined with a single post-label delay (PLD), varying slightly depending on the subject’s age and health status.

Simple transposition of the recommended clinical methods to pre-clinical systems, however, is not sufficient to achieve high quality CBF measurements. Interspecies differences mean that a direct transposition of acquisition parameters yields spurious and error-prone data. These issues pose a significant problem as pre-clinical models of disease are of critical importance in many fields and accurate, non-invasive and quantitative measurement of blood flow is a crucial parameter in many studies. The problems with pre-clinical ASL stem mostly from issues associated with the high field strengths that are used pre-clinically, as well as rodent-specific head and neck geometry. High quality ASL images are dependent upon a uniform magnetic field, not only in the imaging plane, but also in the labelling plane. Off-resonance effects arising from poor magnetic field homogeneity and neck geometry lead to differing labelling efficiency in each vessel in the labelling plane. These differences in labelling efficiencies lead to errors in the calculated CBF, that cannot be corrected from the matched pairs of label and control images acquired during a pCASL MRI experiment. This problem is exacerbated in pre-clinical imaging studies for two reasons: (1) the air spaces in the rodent head (throat, oesophagus, mouth, and nasal cavities) are very close to the labelling and imaging planes, causing large magnetic susceptibility artefacts and off-resonance effects; and (2) the higher field strengths used (typically ≥7 T) make it more difficult to create a homogenous magnetic field. As a result of the above issues, a wide range of CBF measurements in rodents have been reported, often spanning ranges that are neither physiologically realistic (e.g. >300 mL/100 g/min^6–10^), nor in agreement with gold-standard autoradiography measures of perfusion.^11^ Thus, there is a need to improve the application of ASL MRI in rodents and to achieve a similar standardization to that now implemented clinically.

Multiphase pCASL (MP pCASL) is a variant of ASL where instead of acquiring the traditional label and control images, images are acquired following labelling with radio-frequency (RF) pulses at multiple phase increments.^12^ These increments span 360° allowing fitting of the data to an expected function, rather than simply subtracting label from control. These extra phase increments make the acquisition of MP pCASL more time consuming than a simple label-control experiment. However, in a rodent, where the exact magnetic environment in the labelling plane is unknown and likely to be inhomogeneous, the MP pCASL acquisition has the potential to enable post hoc correction for the off-resonance effects in the neck and return true values for CBF. Thus, MP pCASL offers a way to trade increased scan time for a markedly increased confidence in CBF values in rodents. An alternative to MP pCASL, proposed recently by Hirschler et al*.*,^13^ attempts to solve the off-resonance problem through the use of dedicated pre-scans. The proposed approach uses large voxels across each hemisphere, with a high SNR, and then rapidly iterates through a substantial number of phase offsets. These pre-scans are used to determine optimal acquisition parameters for the following ASL acquisition. The drawback with this approach is that where adjacent arteries have different phase offsets, a single value must be chosen for both if using a pre-scan approach, whereas the optimal value may be used for each artery with a post hoc approach. Additionally, the pre-scans must be processed while the rat is still anaesthetised, instead of a complete post hoc processing that is possible with a full MP pCASL acquisition.

The primary aim of this study, therefore, was to implement, optimise and validate an MP variant of pCASL in rats, in order to improve the accuracy and reliability of rodent CBF measurements via post hoc correction for optimal phase. This study was performed in three different strains of rat (Wistar, Sprague Dawley (SD) and Berlin Druckrey IX (BDIX)) to enable robust assessment of the reliability and applicability of the MP pCASL method. CBF measurements are compared to those obtained by ^14^C-iodoantipyrine autoradiography, the pre-clinical gold-standard technique for quantitative CBF determination.^11,14^

## Materials and methods

### Animals

Female Wistar, SD and BDIX rats weighing 180–400 g were used throughout. All animal experiments were approved by the UK Home Office (Animals [Scientific Procedures] Act 1986) and conducted in accordance with the University of Oxford Policy on the Use of Animals in Scientific Research and the ARRIVE guidelines.^15^ Animals were housed in individually ventilated cages under a 12-h light/12-h dark cycle with food and water ad libitum. All animals were housed in cages of three or four animals, depending on body mass.

### Carotid blood flow measurement

Rats (*n* = 3 per strain) were anaesthetised with isoflurane and laid supine on an ultrasound stage. Body temperature was maintained at *ca.* 37℃ using a homeothermic pad under the animal. Each animal’s carotid arteries were observed by ultrasound for 30 s at 10–12 respiration rates at 39–65 breaths per minute. B-mode ultrasound videos were acquired and used to determine the time-averaged mean and peak blood velocities at each respiration rate (Figure SI1 and Supplementary Methods for full details).

### Simulations of ASL

Numerical simulations using the Bloch equations were conducted using previously developed code^16^ in Matlab (Mathworks, Natick, MA) to determine theoretical blood inversion during the ASL pulse sequence (i.e. M(z), expressible as labelling efficiency – the percentage of maximum inversion possible). A pCASL sequence was simulated with a pulse train of 600 µs Hanning-shaped pulses, each separated by 600 µs. Simulated T_1_ and T_2_ of blood was 2.1 s and 33 ms, respectively, measured from oxygenated rat blood at 37℃. The ratio G_max_/G_av_ during labelling was fixed at 20. Blood velocity was simulated from 1 to 100 cm/s, labelling gradient strengths were varied to correspond to a labelling plane thickness from 2 to 10 mm and labelling pulse flip angle varied from 2 to 90°. The relationship between G_max_ and labelling plane thickness (*Thk_lab_*) is given by *Thk_lab_* = *BW_trans_*/(*γ_0_*·*G_max_*), where *BW_trans_* is the transmitted RF bandwidth (4.3 kHz) and *γ_0_* is the gyromagnetic ratio (42.58 MHz/T).

### Magnetic resonance imaging

Rats were anaesthetised with isoflurane and imaged using a 9.4 T MRI spectrometer (Agilent) with a 72 mm volume transmit coil and a 4-channel surface receive array (Rapid Biomedical; *n* = 7 per strain). Respiration rate was maintained between 40 and 60 breaths per minute by adjusting isoflurane concentration.

**Figure 1.**
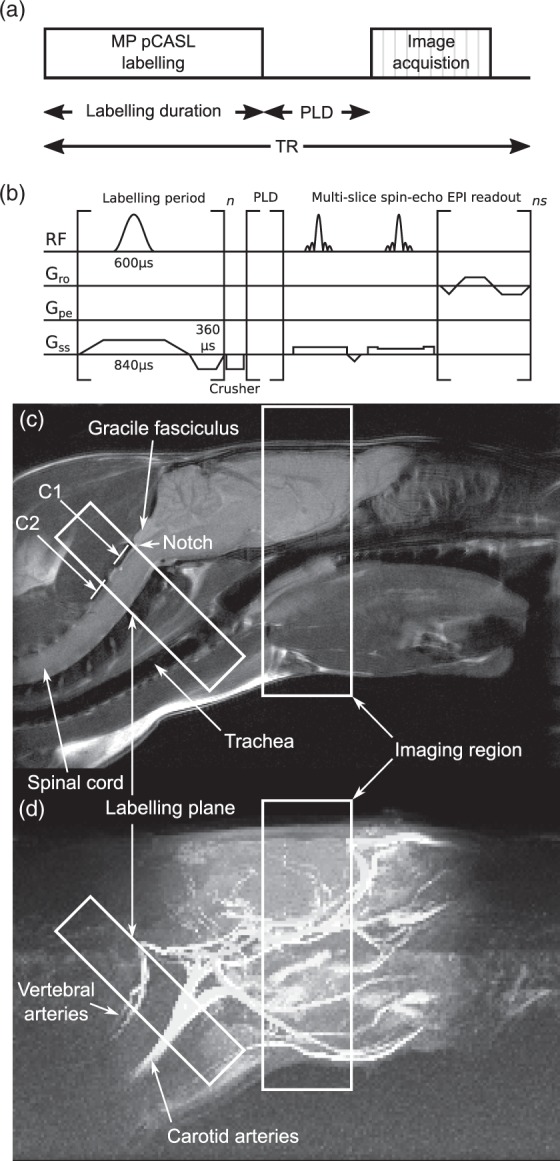
(a) Schematic of the multiphase pCASL sequence. (b) Pulse timing diagram of the sequence. (c and d) Location of the labelling and imaging regions shown in relation to the brain and the major vessels of the neck, superimposed over (c) an anatomical, fast spin-echo sagittal midline image of an SD rat head, and (d) a maximum intensity projection of time-of-flight angiography with the same field of view. Positions of C1 and C2 vertebra are visible and the notch immediately caudal to the gracile fasciculus is easily identified.

A multiphase pCASL sequence^12^ was implemented by varying the phase increments of pulses in the labelling train from 0° to 315° in eight steps of 45°. The labelling plane (6.2 mm thick) was placed in the neck of the rat, either perpendicular to, or at 45° to, the animal’s rostro-caudal axis. The location of the labelling plane was set through the use of a midline sagittal fast spin echo image (FOV = 50 × 50 mm, matrix = 256 × 256, thickness = 2 mm, TR = 1 s; TE_eff_ = 40 ms, T_exp_ = 34 s, single scan). For the MP pCASL, a multislice single-shot spin-echo echo planar imaging (EPI) sequence was used for the imaging readout (FOV = 32 × 32 mm, matrix = 64 × 64, thickness = 1 mm, 10 slices, TE = 28.7 ms). Slices were acquired in an anterior-posterior direction. Blood labelling (tagging) was achieved with a pulse train comprising Hanning-shaped pulses of 600 µs duration and 40° flip angle, each separated by 600 µs (50% duty cycle). A schematic of the pulse sequence is shown in Figure 1. Proton-density calibration images for absolute CBF quantitation were acquired for all animals, using both the surface receive array and the volume transmit coil, by omitting labelling pulses. For all animals, T_1_ maps were obtained using an inversion recovery method (inversion time varied in nine logarithmic steps from 0.013 to 8 s, TR = 10 s) and T_2_ maps were obtained using a multi-echo approach (echo time varied in nine logarithmic steps from 30 to 160 ms, TR = 10 s). Readouts were spin-echo EPI acquisitions as for the ASL acquisitions.

Arterial transit time (ATT) was measured by acquiring data with 12 PLDs (10, 15, 20, 25, 30, 50, 100, 200, 300, 500, 750 and 1000 ms; PLD), each with the same eight phase angles (acquisition time = 14 min 41 s for all 12 PLDs combined). The spacing of PLDs was chosen to obtain good fits for typical BATs observed in pilot data. Multi-PLD images were acquired with only two slices – in naïve animals, the first immediately posterior to the olfactory sulcus (i.e. most anterior slice of the full imaging region) and the second 10 mm posterior to the first (i.e. most posterior slice of the imaging region); in tumour-bearing animals, the slices were positioned across the tumour. This reduction in imaging slices was necessary as each slice took 50 ms to acquire and obtaining 10 slices would lead to an unacceptable minimum delay before acquiring the last slice.

In a subset of animals also used for autoradiography (*n* = 3 per strain), more extensive optimisations were carried out. Label duration was varied as follows: 0.4 s, 0.9 s, 1.4 s (TR = 4 s, T_exp_ = 1 min 29 s), 2.4 s (TR = 5 s, T_exp_ = 1 min 39 s), 3.7 s (TR = 6.3 s, T_exp_ = 2 min 4 s), 5.0 s (TR = 7.6 s, T_exp_ = 2 min 30 s), all with PLD = 550 ms. Label location was varied serially along the neck vasculature in 2 mm increments. To confirm labelling plane location with respect to the brain and brain vasculature, time of flight (TOF) angiography was used to visualise vessels and brain-midline sagittal anatomical MRI was used to show the labelling plane location with respect to the vessels. TOF angiography: T_1_-weighted 3D gradient echo readout (FOV = 40 × 40 × 60 mm, matrix = 128 × 128 × 192, TR = 30 ms, FA = 30°, axial excitation slab, T_exp_ = 12 min 17 s). Anatomical MRI: T_2_-weighted fast spin-echo readout (FOV = 40 × 60 mm, matrix = 128 × 192, TR = 1 s, TE_eff_ = 10 ms, single 2 mm slice at brain midline, T_exp_ = 2 min 8 s).

T_1_ and T_2_ times of re-oxygenated post-mortem blood were determined at 37℃, using a heated water jacket sample holder to maintain the temperature. Oxygenation was confirmed as complete using an i-STAT blood-gas analyser (Abbot, UK).

### Data fitting and analysis

**Figure 2.**
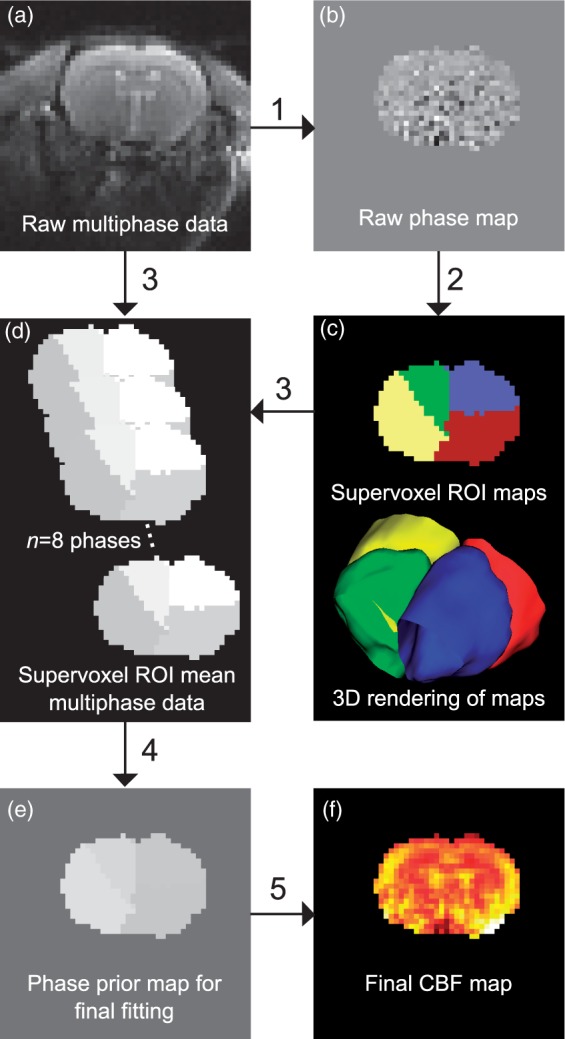
Schematic showing the use of supervoxels in preparing high precision phase map priors. The raw multiphase data (a) are initially fitted to the Fermi function with low precision priors (1) to yield a raw phase map (b). This raw phase map contains good spatial information but the phase values themselves are overestimated. The data are smoothed and clustered using supervoxels (2) to yield ROIs for each supervoxel phase cluster present (c). The raw multiphase data are then combined with the supervoxel ROIs (3) to yield high SNR supervoxel-ROI means of the multiphase data (d). This high SNR multiphase dataset is then fitted again (4) yielding a high precision phase map (e) which can be used to generate the final CBF maps (f), in combination with the raw data (5).

ASL data analysis and perfusion quantitation were performed using a custom version of BASIL,^17^ which is available as part of the FMRIB Software Library (www.fmrib.ox.ac.uk/fsl/basil). The multiphase acquisition data are expected to fit a modified Fermi function of the form

$$f(\mathrm{phase})=\mathrm{Mag}(-2[\frac{1}{1+e^{(|\mathrm{phase}|-\alpha )/\beta }}])+\mathrm{Off}$$

where *α* and *β* were 70 and 19, respectively; chosen by minimisation of root-mean-square error on data fitting of 12 rats from three strains (data not shown), *phase* is the phase angle in degrees, *Mag* is the magnitude (amplitude) and *Off* is the offset from 0.^12^ The Fermi function was fitted to the multiphase data to obtain the magnitude of the multiphase ASL signal. Fitting was performed using the Variational Bayesian algorithm within the FSL tool fabber, which incorporates normally distributed priors on all the parameters.^17^ Since 3-parameter models such as this are prone to over-estimation of amplitude in the presence of additive noise,^18^ a multi-step analysis process was used to minimise bias. Firstly, the raw multiphase data were fitted with unconstrained priors, producing voxel-wise estimates of phase, magnitude and offset. The phase maps were clustered using a supervoxel approach^19^ with four supervoxels per phase map, a smoothing (σ) of 0.8 and a compactness of 0.1. This was used to define ROIs from the data that represent individual flow territories as indicated by their phase value arising from the phase offset present in the feeding artery. For each supervoxel region, the original multiphase data were averaged for all voxels across each phase, yielding a new higher SNR multiphase dataset. This dataset was fitted to the Fermi function, estimating a single phase value for each supervoxel ROI. These final phase values were used as a high precision prior for the final fitting of the original raw multiphase data, yielding a voxel-wise map of magnitude and offset. Figure 2 shows a flow chart of this methodology, including example images derived by the process. Datasets from this manuscript are available upon request.

The resultant maps of the magnitude of the fitted function, representing the presence of labelled water in each voxel and equivalent to the subtracted label-control images from traditional pCASL, were processed with oxford_asl (part of the FMRIB software library^17,20^) in the same way as would be done with conventional label-control difference data and according to the one-compartment kinetic model of Buxton et al.^21^ to produce relative CBF maps. In the subset of animals used for autoradiography (*n* = 3 per strain), comparisons to the one-compartment model were made by also fitting the data according to the two-compartment kinetic model of Parkes and Tofts.^22^ This two-compartment model includes an extravascular water pool in addition to the blood compartment water pool and has two described solutions, one ‘slow’ and one ‘fast’. The slow solution assumes that labelled water never leaves the tissue voxel, while the fast solution assumes that venous blood has the same magnetisation as blood in the voxel. Maps were corrected for coil sensitivity inhomogeneity, using the ratio of proton density images acquired with the surface receive array and the volume coil. Final calibration to absolute CBF units was performed using a reference region M0 value method. The striatum was chosen as a reference region since the use of CSF, as used in humans, was not practical in rats owing to its small volume and consequent partial volume effects that prevent selection of voxels containing CSF alone. The rat striatum is large enough to provide sufficient voxels for analysis, is clearly identifiable, and is easy to use for in vivo T_1_ and T_2_ time quantitation. A striatum-specific tissue:blood partition coefficient for water of 0.97^23^ and a single inversion efficiency, 82%, calculated as a mean for the three strains from simulations of each strain-specific carotid blood velocity (see results) was used. Relaxation parameters for quantitation were: blood, T_1_ = 2.09 ± 0.02 s, T_2_ = 33.2 ± 0.3 ms; reference tissue (striatum), T_1_ = 1.5 ± 0.2 s; T_2_ = 40 ± 8 ms; whole brain, T_1_ = 1.6 ± 0.3 s.

For conventional label-control analysis, 0° and 180° images from the multiphase acquisition were used as label and control, respectively. CBF quantitation was carried out using the voxel-wise calculation recommended in the White Paper^5^

$$\mathrm{CBF}=\frac{6000\cdot \lambda \cdot ({\mathrm{SI}}_{\mathrm{control}}-{\mathrm{SI}}_{\mathrm{label}})\cdot e^{\frac{\mathrm{PLD}}{T_{1,\mathrm{blood}}}}}{2\cdot \alpha \cdot T_{1,\mathrm{blood}}\cdot {\mathrm{SI}}_{\mathrm{PD},\mathrm{Str}}\cdot (1-e^{-\;\frac{\tau }{T_{1,\mathrm{blood}}}})}$$

where *λ* is the brain/blood partition coefficient of water (0.9 mL/g), *SI_control_* and *SI_label_* are the signal intensities of the control and label images, PLD is the post label delay (0.55 s), *T*_1,_*_blood_* is the longitudinal relaxation time of rat arterial blood at 37℃ (2.1 s), *α* is the labelling efficiency for pCASL (0.85), *SI_PD,Str_* is the mean signal intensity for an ROI drawn across the striatum of the proton-density-weighted reference image with no labelling pulses, corrected for short TR by multiplying by (1/(1-e^-TR/T1,tissue^) where T_1,tissue_ is the measured longitudinal relaxation time for rat brain (1.6 s), and τ is the label duration (1.4 s).

### Autoradiography

CBF was determined using gold standard autoradiography^11,14^ in all three strains (*n* = 3 per strain). Briefly, rats were anaesthetised and 4[N-methyl-^14^C] iodoantipyrine was infused into the femoral vein over 1 min (50 µCi in 0.5 mL saline). The rat was killed by pentobarbitone infusion, immediately decapitated, and the head frozen. Brain slices (20 µm thickness) were exposed to X-ray film with calibrated images being converted to CBF values. CBF maps were aligned with respective to MRI CBF maps. Whole brain, striatum and cortex ROIs were drawn on the MRI maps and the same ROIs were transferred to the autoradiography images for comparison. Full methodological details are given in the Supplementary Methods.

### Intracerebral model of brain metastasis

Female BDIX rats (*n* = 3) were anaesthetised with isoflurane (1.5–3% in 70:30 N_2_O:O_2_) and placed in a stereotactic frame. An incision was made on the scalp and a burr hole drilled 1 mm anterior and 3 mm lateral to bregma; 1000 ENU1564 cells in 1 µL PBS were injected using a drawn glass capillary (<100 µm diameter) to a depth of 3.5 mm in the left striatum. The scalp was sutured and animals were allowed to recover. Tumour-bearing BDIX rats were subjected to MRI three weeks after tumour induction. Group size was chosen from a power calculation assuming a 30% decrease in blood flow in tumours, with standard deviation equal in both groups to that observed in naïve animals and 1−β = 0.9.

### Statistical analysis

All results are presented as mean ± S.D, unless otherwise specified. Differences between groups were determined using one-way ANOVA followed, where required, by Tukey’s multiple comparison post hoc tests.

## Results

### Angiography and labelling plane orientation

Time-of-flight angiography showed orientation and location of major vessels with respect to head, neck and brain anatomy visible on high-resolution structural images (Figure 1(c) and (d)). No discernible differences in relative geometry were evident between strains of rat or between smaller and larger rats (range from 180 to 340 g). Carotid and vertebral arteries ran at angles of 42 ± 7° and 44 ± 8°, respectively, with respect to the longitudinal axis of the rat and in no strain was there a significant difference in angle between the carotid and vertebral arteries. The bend in the vertebral arteries immediately rostral to the C1 vertebra, and just prior to the entrance into the skull, marks the limit of the region where the vessels are straight and parallel. This point can be seen as the notch immediately caudal to the gracile fasciculus and is easily visible on sagittal midline anatomical images (Figure 1(c)).

The orientation of the labelling plane across the neck impacted both absolute CBF quantitation and error in quantitation of the CBF maps. Flow-driven adiabatic inversion is most efficient when the flow of blood is perpendicular to the labelling plane. A labelling plane at 45° with respect to the longitudinal axis of the animal crosses the arteries close to perpendicularly. The CBF maps obtained at the optimal 45° labelling angle exhibited lower variance in voxel-wise CBF values (*p* < 0.01; F test) and yielded more physiologically realistic absolute CBF values than images acquired with a labelling plane at 0° (*p* < 0.001; *t*-test; Figure SI2(a)). Example of CBF maps is shown in Figure SI2(b).

### Blood velocity measurements

**Figure 3.**
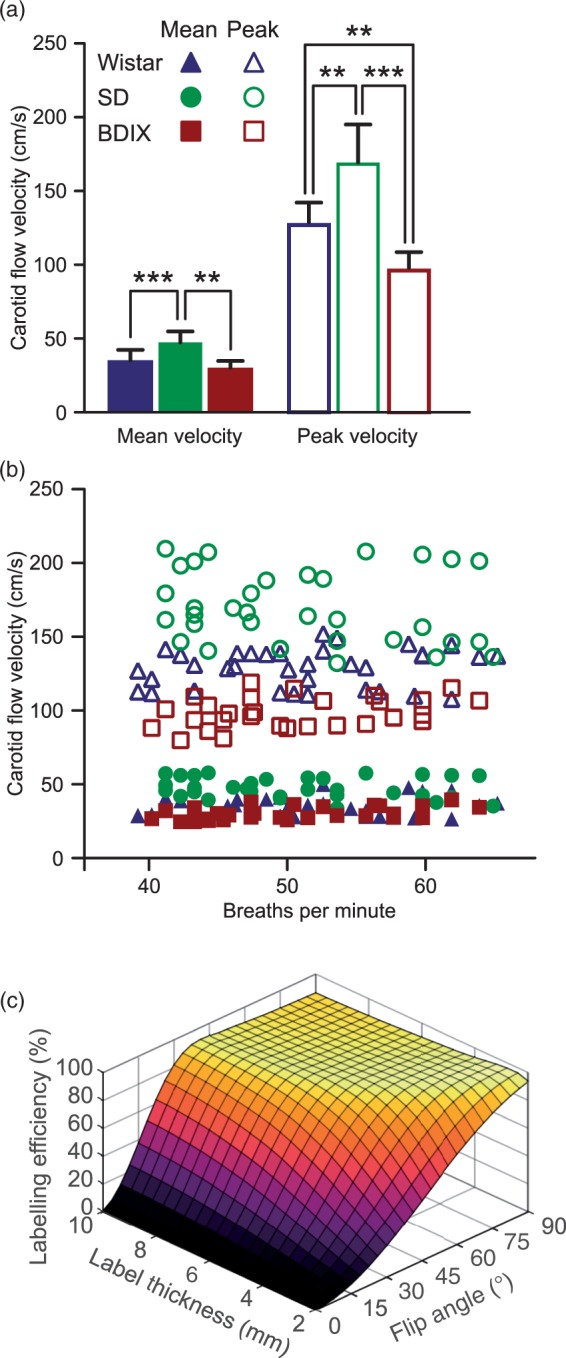
(a) Mean and peak carotid artery flow velocities in three strains of rats; Wistar, SD and BDIX. (b) Mean and peak carotid artery flow velocities as a function of anaesthetic depth, as indicated by breathing rate. (c) Bloch simulation results showing labelling efficiency (inversion achieved as a percentage of theoretical inversion possible), for blood as it passes through labelling planes of 2–10 mm thickness with flip angles in the labelling pulse train between 2° and 90° at 37 cm/s (mean carotid velocity for all rats studied).

Carotid artery blood flow velocities were measured by Doppler ultrasound in Wistar, SD and BDIX rats (*n* = 3 for each strain). Typical acquisition traces and images, from which mean and peak (systolic) flow velocities were determined, are shown in Figure SI1. Mean carotid artery flow velocity was significantly higher in SD rats compared to either BDIX or Wistar rats (47 ± 6 cm/s for SD vs. 30 ± 10 cm/s for BDIX, *p* < 0.001; and 35 ± 7 cm/s for Wistar, *p* < 0.01; Figure 3(a)). Peak carotid artery flow velocity was lowest in BDIX rats (100 ± 30 cm/s), higher in Wistar rats (130 ± 20 cm/s; *p* < 0.01) and higher again in SD rats (170 ± 20 cm/s; *p* < 0.01). No significant difference was evident in either peak or mean flow velocity as a function of respiration rate within any strain (Figure 3(b)).

### Simulation results

Numerical simulations showed that inversion efficiencies diminished dramatically with low flip angles (<30°), especially with narrow labelling planes and/or high blood velocities. However, there exists a plateau of high and relatively uniform inversion efficiency at greater labelling region thicknesses and flip angles (Figure 3(c)). A labelling region thickness of 6.2 mm and a flip angle of 40° was selected for in vivo experiments to achieve maximum inversion efficiency across a range of physiologically relevant blood velocities (25–50 cm/s; Figure SI3). No significant difference in inversion efficiencies was evident between strains, despite the higher mean blood velocity in the SD rats, which is a result of the non-linear relationship between velocity and inversion efficiency (Figure SI3). Mean inversion efficiency for the three strains of rats was 82%.

### Relaxometry

Whole, 100% oxygenated rat blood relaxation times at 37℃ and 9.4 T were: T_1_ = 2.09 ± 0.02 s; T_2_ = 33.2 ± 0.3 ms. Striatal relaxation times *in* vivo were: T_1_ = 1.5 ± 0.2 s; T_2_ = 40 ± 8 ms. Whole brain in vivo T_1_ relaxation time was 1.6 ± 0.3 s (*n* = 21 rats across 3 strains). These values were used to calibrate quantitation procedures as described in the ‘Methods’.

### PLD and arrival time

**Figure 4.**
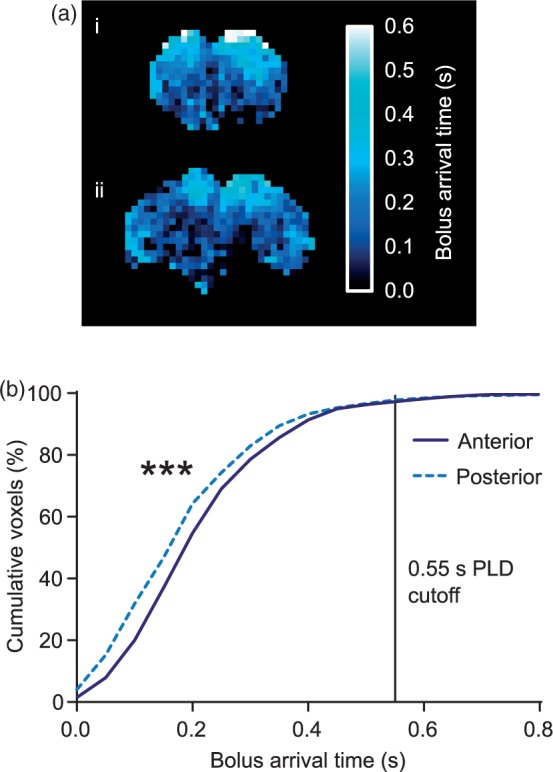
(a) Bolus arrival time maps from (i) anterior slice (immediately caudal to the olfactory sulcus), and (ii) posterior slice (10 mm caudal to the anterior slice) from an example BDIX rat brain. (b) Cumulative frequency distributions of voxel arrival time in the anterior and posterior slices from all rat strains (*n* = 18; ***p < 0.001). The PLD cut-off represents the point at which arterial transit (cumulative voxels) had occurred in 97% of voxels imaged.

Images acquired with varying PLDs, between 10 and 1000 ms, were used to construct blood bolus arrival time maps (Figure 4(a)). Evaluation of arrival time in two slices, one at the front and one at the back of the imaging region within the brain, allowed the range of possible arrival times to be determined. Example of multi-PLD data for whole animals and example of single voxels are shown in Figure SI4. As expected, blood arrived at the anterior slice later than it arrived at the posterior slice in all strains (*p* < 0.001, Kolmogorov–Smirnov test; Figure 4(b)), but a large overlap was evident between the range of arrival times at the front and back of the brain. A PLD of 550 ms was chosen for subsequent imaging as 97% of voxels in both slices had an arrival time shorter than this. For tumour-bearing BDIX rats, mean arrival time for voxels within the tumour was slower for those voxels in a comparable contralateral ROI (Figure SI5, *p* < 0.001). CBF was heterogeneous across the tumour area and where CBF in the tumour was greater than the minimum CBF of the contralateral ROI (i.e. excluding voxels in core hypoxic regions of the tumour), >87% of tumour voxels had an arrival time of ≤650 ms. We therefore used a PLD of 650 ms for tumour-bearing animals. Voxels in the core of the tumour where CBF was very low had longer arrival times (up to 1600 ms).

### Labelling plane location and labelling duration

**Figure 5.**
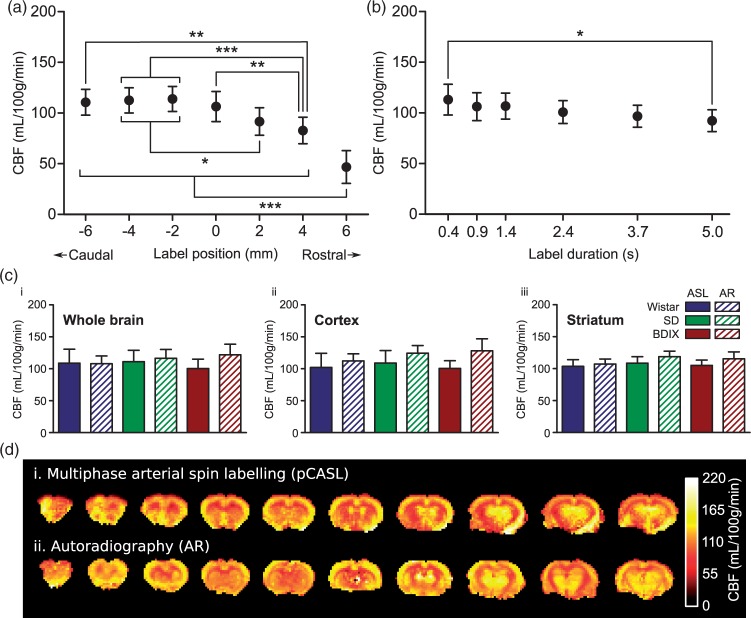
(a) Effect of labelling plane location on CBF measurements. Negative label positions are located towards the tail of the animals, positive positions towards the nose; 0 mm is the position of the labelling plane shown in Figure 1, at which the labelling plane is entirely spanning straight and parallel vessels, each passing close to perpendicular through the labelling plane. ***p < 0.001, **p < 0.01, *p < 0.05; *n* = 9 across three strains. (b) Effect of label duration on CBF measurements: *p < 0.05; *n* = 9 across three strains. (c) Comparison between autoradiography (AR) and pCASL (1.4 s label duration) derived CBF values from ROIs covering (i) the whole brain, (ii) the cortex, or (iii) the striatum. (d) Example CBF maps obtained using (i) multiphase pCASL MRI and (ii) autoradiography in a Wistar rat.

The position of the labelling plane was moved along the axis of the vessels in the neck over a range of 12 mm. The extent of this range was ultimately limited by interference with the imaging plane at the rostral end and by efficiency of the labelling coil (volume transmit RF coil) at the caudal end. However, before reaching the rostral limit, the labelling plane moves across regions where the feeding arteries have entered the brain and no longer run perpendicular to the labelling plane; for example, as the vertebral arteries enter the skull, blood flows parallel to the labelling plane (Figure 1(d)). These imperfections affect labelling efficiency and lead to artificially decreased CBF values (Figure 5(a)). As the labelling plane was moved caudally from this point, through the region where the feeding vessels are straight and parallel, no significant effect on calculated CBF values was found.

No significant differences were found between CBF values obtained at any label duration from 0.9 s to 3.7 s. However, a significantly lower CBF was obtained with the longest label duration of 5 s compared to the shortest duration of 0.4 s (113 ± 15 vs. 92 ± 11 mL/100 g/min, *p* < 0.05; Figure 5(b)). SNR efficiency (expressed as *accumulated signal/√T_exp_*) exhibits a sharp increase up to a label duration of 1.4 s, then remains broadly similar with label durations between 1.4 s and 5 s, albeit with a peak at around 2.4 s (Figure SI6). For all subsequent acquisitions, a label duration of 1.4 s was used to reduce scan time.

### Observed phase offsets within and between animals

Optimal phase offsets varied both between and within animals, with greater variation between animals. Within animals, the range of optimal phases between supervoxel regions varied from 6 to 107° (median 21°, first and third quartiles: 11° and 31°). Between animals, the absolute phase offset was distributed widely across the complete 360° (a graphical plot of all phase offsets for all supervoxels is shown in Figure SI7). No correlation was evident between mean phase offset for an individual animal and the dispersion of phase angles is observed between supervoxel territories for that animal. Moving the labelling plane within the neck of an animal was sufficient to elicit significant changes in optimal phase angles observed in some, but not all, cases, likely reflecting the unique magnetic environment in each animal’s neck.

### Model fitting comparison and correlation between pCASL and autoradiography CBF values

No significant differences were found in any region (cortex, striatum or whole brain) or strain between CBF values obtained by autoradiography and multiphase pCASL MRI when fitted with the one-compartment model.^21^ Representative pCASL MRI data acquired with a label duration of 1.4 s are shown in Figure 5(c), but no differences between pCASL MRI and autoradiography CBF measures were found at any label duration from 0.9 s to 3.7 s. Autoradiography values for whole brain perfusion were 108 ± 12, 116 ± 14 and 122 ± 16 mL/100 g/min in Wistar, SD and BDIX rats, respectively, compared to multiphase pCASL CBF values in the same animals of 109 ± 22, 111 ± 18 and 100 ± 15 mL/100 g/min, respectively, with the one-compartment model. Mean CBF values across all animals (*n* = 9) obtained using autoradiography (115 ± 14 mL/100 g/min) and multiphase pCASL analysed using the one-compartment model (107 ± 13 mL/100 g/min) were not significantly different. In contrast, the CBF values from the two implementations of the two-compartment model were both significantly different to the autoradiography CBF measures (95 ± 11 mL/100 g/min for the slow implementation, *p* < 0.05; and 157 ± 19 mL/100 g/min for the fast implementation, *p* < 0.001; one-way ANOVA with Dunnett’s test). CBF values obtained using the one-compartment and slow two-compartment models were not significantly different.

### Multiphase analysis vs. label-control analysis

**Figure 6.**
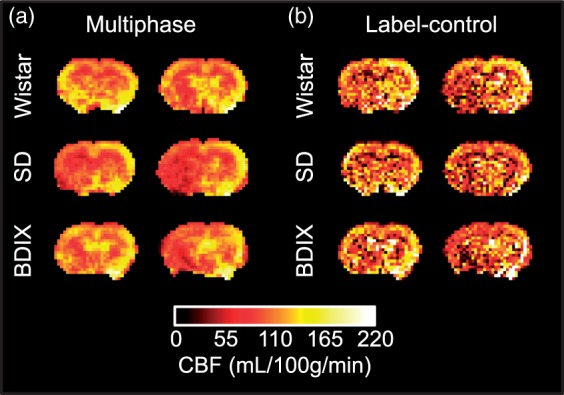
Comparison of CBF maps from single-average multiphase pCASL acquisitions and four-average label-control acquisitions in the same animal; total imaging time 89 s in each case. Note the lower CBF values, areas of greater heterogeneity and the regions with decreased apparent perfusion in the label-control maps.

A comparison between multiphase analysis and the White Paper-recommended label-control analysis was made for each animal. Label-control images (effectively two phases) can be acquired in one-quarter the time of the eight-phase multiphase acquisitions, and therefore, four averages were used for the label-control acquisitions for legitimate comparison. Label-control subtractions gave images with lower CBF values (*p* < 0.01) and higher standard deviations (F-test, *p* < 0.01). Additionally, some regions appeared hypo-perfused in the label-control images, but multiphase images showed that they were in fact normally perfused (Figure 6).

### Strain-specific CBF measurements and brain metastasis model

**Figure 7.**
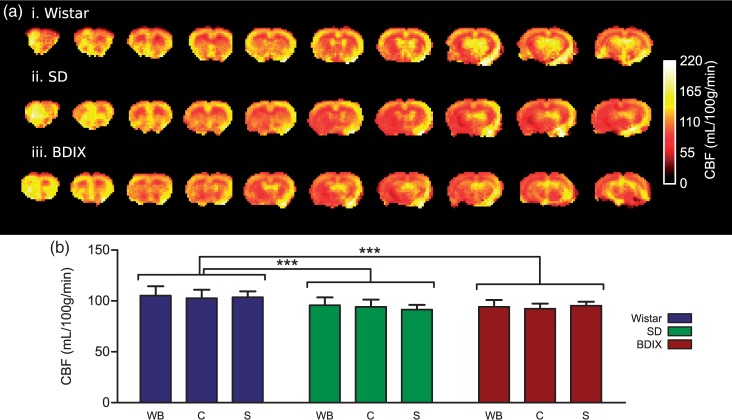
(a) Example cerebral blood flow maps acquired using the optimised multiphase pCASL sequence for three strains of rat; Wistar, SD and BDIX. Eight averages were acquired per image, total imaging time = 11 min 52 s. (b) CBF mean of whole brain (WB), cortex (c), and striatum (S) in three strains of rats. *n* = 7 rats/strain; *p < 0.05.

Having validated the optimised multiphase pCASL parameters through comparison of CBF values with gold standard autoradiography, a larger cohort of each strain (*n* = 7 per group, including the three autoradiography animals) were subsequently used to assess reproducibility of the multiphase pCASL measurements. Example of strain-specific maps of multiphase pCASL CBF values is shown in Figure 7. Wistar rats had a significantly higher CBF than the other two strains (*p* < 0.001). However, within each strain, no significant differences in CBF were found between ROIs studied. Whole brain CBF varied very little between strains, being 106 ± 17, 96 ± 22 and 95 ± 10 mL/100 g/min in Wistar, SD and BDIX rats, respectively. These values show good correspondence with those obtained in the autoradiography experiment. In tumour-bearing rats (*n* = 3), blood flow was decreased to 60 ± 5 mL/100 g/min in an ROI covering the tumour area from 93 ± 3 mL/100 g/min in a comparable contralateral ROI (*p* < 0.001, Figure SI5).

## Discussion

In this work, a multiphase pCASL MRI technique was optimised for pre-clinical studies in rats. We have identified imaging parameters that address the issues associated with high magnetic field strength imaging in rodents. The single largest factor in improving image quality was the implementation and use of the multiphase pCASL technique instead of a simple label-control sequence. Choice of imaging parameters was validated by comparison of rat CBF values obtained by multiphase pCASL to gold-standard autoradiography in the same animals. Reproducible and robust CBF measurements were demonstrated using these optimised parameters and methodology.

### Multiphase pCASL vs. label-control ASL

In this work, we used a pCASL implementation for MRI CBF data acquisition, which is the clinically recommended method since it does not require continuous RF transmitting hardware and reduces magnetization transfer effects inherently present in the earlier implemented CASL approach. Moreover, pCASL retains the well-defined bolus duration present in CASL, essential for accurate CBF quantification, while maintaining a higher SNR than PASL.^5,24^ Finally, as with CASL, blood labelling occurs in a narrower plane by flow-driven adiabatic inversion. We also implemented a multiphase approach, rather than the traditionally used single label-control sequence, and this yielded the greatest increase in signal-to-noise and image quality. While the multiphase pCASL acquisitions take up to four times longer than traditional label-control pairs to acquire, the total imaging time of 89 s for an eight phase acquisition is not excessive and yields images that show a considerable qualitative and quantitative improvement over acquiring four averages of label-control subtraction in the same time frame. Note, also, that some regions in the label-control analysis have artificially reduced CBF values that affect entire vascular territories. This result is a consequence of off-resonance effects such that one or more vessels running through the labelling plane are affected differently to the other vessels, and this cannot be corrected without the multiphase data.

### Labelling plane parameters

The ideal labelling plane should be positioned at a point where the traversing arteries are both straight, and angled to be approximately perpendicular to the direction of flow in the vessels. If both criteria are met, the exact distance of the labelling plane from the imaging region is less important. However, for consistency, one location should be chosen for all animals. This point could be determined on an animal-by-animal basis through the use of angiography, but this scan can be avoided through the use of an anatomical reference, as suggested by Alsop et al.^5^ Thus, in rodents, our data suggest that optimal positioning can be achieved by placing the labelling plane immediately caudal to the marked bends in the vertebral arteries, just behind the gracile fasciculus and to the rear of the top of the atlas vertebra (as shown in Figure 1). This anatomical marker is readily identifiable on quick sagittal midline scans and does not vary between rat strains, making it an ideal location.

Corollaries to the position of the labelling plane are its orientation and the label duration. The selection of a 45° labelling plane, instead of a 0° plane, decreases error associated with CBF measurements and, importantly, brings the magnitude of the values closely into alignment with the autoradiography data. With regard to the duration of the labelling period, ASL signal increases as label duration increases, albeit with a plateau at longer labelling times (*signal* ∝1-(*e^-^*^(label duration/T1)^). The efficiency of accumulated signal is unacceptably low with label durations <1.4 s but broadly similar with label durations from 1.4 s to 5 s, giving investigators the option to vary label duration (and thus experimental acquisition time), to best align with their experimental goals. The shorter acquisition for the 1.4 s label duration offers the better compromise in our current application (Figure SI6).

One important aspect of the analysis pipeline is the selection of the correct phase priors for model fitting. It is a mathematical problem that three component models, with a phase, amplitude and offset (such as the Fermi function used herein), are prone to overestimation of the amplitude when noise is increased.^18^ Noise in the multiphase pCASL signal is increased with short label durations as there is less labelled blood in the imaging plane, meaning that changes in image intensity are closer to acquisition and biological noise floors. A consequence of this effect is that, without steps to choose the correct phase for data, the magnitude (and hence CBF) is overestimated (data not shown). By using the supervoxels to cluster phase maps (predominantly within vascular territories in the brain), then averaging the voxels in the original multiphase pCASL data within each ROI, the SNR of the raw data was increased at the expense of spatial resolution. This approach can be justified since phase estimates are not expected to vary on a voxel-wise basis within whole vascular territories. By using the supervoxel approach, higher precision fitting was possible, yielding more accurate phase values and improved magnitude fitting results. Consequently, the relationship between label duration and CBF is broadly flat using this approach, without overestimation of CBF at the shorter label durations. Despite these corrections, however, a slight trend for increased measured CBF is still seen at the shortest label durations, probably an artefact of the very poor ASL data achieved with such short label durations. In contrast, a trend towards decreased CBF at the longest label durations is evident, which may be either a consequence of inaccurate T_1_ estimates or a consequence of not considering outflow of labelled blood from the tissue during the labelling period. In humans, this outflow is considered negligible, but in rats may confer a moderate contribution at long labelling times.

### Arrival time and PLD selection

A short PLD is advantageous for maximising the ASL signal as it minimises T_1_ recovery of the ASL signal from the labelled water in the imaging region. However, if the PLD is too short, the transit of labelled blood through the arteries and into the tissue will be incomplete: this can lead to signal from arterial blood artificially increasing the measured CBF values near arteries and underestimation of tissue CBF elsewhere. In this study, the BAT in naïve rats was <550 ms in 97% of voxels, independent of strain. Consequently, a PLD of 550 ms was chosen as the best compromise for maximum signal without arterial contamination. However, in the case of pre-clinical models of brain pathology, particularly where vascular changes are expected to occur, such as stroke or cancer, assessment of BATs in representative animals would be necessary to determine an appropriate PLD for imaging, or multi-PLD protocols could be considered. For example, in our tumour-bearing BDIX rats, we chose a longer PLD to compensate for the later arrival exhibited by tortuous tumour vessels. In all cases, this choice will be a compromise between increasing PLD to accommodate voxels with a later arrival time and decreasing PLD to prevent too much signal decay by T_1_ relaxation.

### Validation with gold standard measurements and model comparisons

Autoradiography measurements represent the gold standard in brain perfusion quantitation and have a long literature validation behind them.^11,14,25^ However, autoradiography is an invasive and terminal procedure, as well as being complex and time consuming to perform and quantify. These factors render autoradiography unsuitable for large numbers of studies, but it remains an excellent calibration point for non-invasive measurements such as pCASL MRI. Here, we compared the autoradiography CBF measurements with CBF values produced by one- and two-compartment kinetic models. The range of the CBF values produced from the three models likely represents an outside limit to the true CBF value, which the autoradiography measurements suggest falls at the lower end of the range. The fast model may be over-estimating CBF because it does not incorporate a complete venous outflow term, which is likely to be more important as flow rates increase. The one-compartment model and the slow implementation of the two-compartment model are in closer agreement and have both been used pre-clinically previously.^8^ Our results demonstrate that the one-compartment model was the closest match to the autoradiography data and, combined with the optimised imaging parameters, produced consistent CBF values from both naïve and diseased rats.

### Strain comparison

Three pre-clinically relevant strains of rats were used throughout this work to assess the reliability of the method across different strains and to determine whether strain specific differences could be observed. Comparing between strains, Wistar rats had significantly higher CBF than SD and BDIX rats, while SD rats exhibited a higher mean carotid blood velocity than the other two strains. Despite this difference in velocity, no significant difference in inversion efficiency was observed between the strains. Thus, we used a single inversion efficiency of 82% for all rat strains, which eliminates the need to determine strain-specific carotid artery velocities for accurate quantitation. We did not observe any differences in blood arrival time to the brain between strains, meaning that a single PLD is suitable for all strains of naïve rat.

## Conclusions

This paper proposes optimised parameters for ASL in rats with the aim of improving and standardising quantitative ASL in high magnetic field pre-clinical settings. A multiphase pCASL approach was employed, in which images are acquired at multiple phase angles instead of the traditional label-control (0˚–180˚) technique, allowing correction for inevitable off-resonance effects and enhancing image quality in both naïve animals and in a brain tumour model system. The optimal labelling plane position and label parameters for efficient blood inversion, the optimal labelling duration for maximum efficiency of data accumulation, and optimal PLD for minimisation of contamination from arterial signal in CBF maps have been determined. Using gold-standard autoradiography, it was confirmed that the optimised multiphase pCASL method yields accurate CBF values and, thus, provides a rapid and reproducible method for non-invasively measuring CBF in rats.

## Supplemental Material

Supplemental material for Quantitative blood flow measurement in rat brain with multiphase arterial spin labelling magnetic resonance imaging Click here for additional data file. Supplemental material for Quantitative blood flow measurement in rat brain with multiphase arterial spin labelling magnetic resonance imaging by James R Larkin, Manon A Simard, Alexandre A Khrapitchev, James A Meakin, Thomas WOkell, Martin Craig, Kevin J Ray, Peter Jezzard, Michael A Chappell and Nicola R Sibson in Journal of Cerebral Blood Flow & Metabolism

## Supplemental Material

Supplementary figure -Supplemental material for Quantitative blood flow measurement in rat brain with multiphase arterial spin labelling magnetic resonance imaging Click here for additional data file. Supplemental material, Supplementary figure for Quantitative blood flow measurement in rat brain with multiphase arterial spin labelling magnetic resonance imaging by James R Larkin, Manon A Simard, Alexandre A Khrapitchev, James A Meakin, Thomas WOkell, Martin Craig, Kevin J Ray, Peter Jezzard, Michael A Chappell and Nicola R Sibson in Journal of Cerebral Blood Flow & Metabolism
