# Supplementary material for: Quantitative blood flow measurement in rat brain with multiphase arterial spin labelling magnetic resonance imaging
Source: J Cereb Blood Flow Metab. 2018 Mar 2;39(8):1557–69. doi: 10.1177/0271678X18756218 (PMC6681434; doi:10.1177/0271678X18756218)
Supplement: Supplemental material for Quantitative blood flow measurement in rat brain with multiphase arterial spin labelling magnetic resonance imaging [file Supplemental_material.pdf]

Supplemental methods to accompany "Quantitative blood flow measurement in rat brain with multiphase arterial spin labelling magnetic resonance imaging"

James R. Larkin[*], Manon A. Simard[*], Alexandre A. Khrapitchev[a], James A Meakin[b], Thomas W. Okell[b], Martin Craig[c], Kevin J. Ray[a], Peter Jezzard[b], Michael A. Chappell[c], Nicola R. Sibson[a]

[a]CRUK & MRC Oxford Institute for Radiation Oncology, Department of Oncology, University of Oxford
[b]Wellcome Centre for Integrative Neuroimaging, FMRIB Division, University of Oxford, Oxford
[c]Institute of Biomedical Engineering, University of Oxford, Oxford

Carotid blood flow measurements by ultrasound

Rats (*n*=3 per strain) were anaesthetised with isoflurane and laid supine on an ultrasound stage. Body temperature was maintained at *ca.* 37ºC through use of a rectal temperature probe and an electrically-heated blanket under the animal. Hair across the neck was removed by clipping and depilatory cream. An MX550D probe coupled to a Vevo 3100 ultrasound (Visualsonics) was used in B-mode and parallel to the rostro-caudal axis to visualise the arteries in the neck. The angle of the carotid arteries with respect to the probe was measured (Figure SI1) and the blood velocity calculated using $f_{\text{doppler}}=(2\cdot f_0\cdot v_{\text{blood}}\cdot \cos\,\theta)/c$, where f_{doppler} is the Doppler frequency, f_0 is the transmitted ultrasound frequency, v_{blood} is the blood velocity, c is the speed of sound in tissue (1540m/s) and θ is the angle between the probe and the blood vessel. Each animal was observed for 30 seconds at 10-12 respiration rates between 39 and 65 breaths per minute. Acquired videos were used to determine the time-averaged mean and peak blood velocities at each respiration rate.

^{14}C-iodoantipyrine autoradiography

CBF was determined using gold standard autoradiography[1,2] in all three strains (*n*=3 per strain). Rats were anaesthetised with isoflurane (4% induction, ~2% maintenance) and the femoral artery, vein and a tail vein were cannulated. 4[N-methyl-^{14}C] iodoantipyrine (Hartmann Analytic, Germany, specific activity: 55mCi/mmol) was infused at a linear continuous rate into the femoral vein over 1 minute (50µCi in 0.5mL saline). Arterial blood was removed from the animal at the same rate as infusion to maintain a constant blood volume. A pentobarbitone overdose was infused into the tail vein after one minute, followed immediately by decapitation and freezing of the head in isopentane on dry ice. The brain was removed from the frozen head and slices (20µm thickness) were dried on a hot plate (60ºC) for 10 minutes, exposed to X-ray film with a calibrated standard for three days (film: Carestream Kodak BioMax MR; standards: 0-35µCi/g, ARC; scanner: Expression 10000XL transmittance scanner, Epson, UK). Scanned films were background-subtracted and calibrated against the standards, before conversion to absolute CBF using and the following equation:

$$C_i(T) = \lambda K \int_0^T C_A e^{-K(T-t)} dt$$

where C_i is the concentration of the tracer at final time (T), λ is the tissue:blood partition coefficient, C_A is the concentration of the tracer in the artery at time t, and K is a constant that incorporates the rate of blood flow into the tissue ($K=mF/W\lambda$, where m is the diffusion equilibrium between blood and tissue (assumed to be 1) and F/W is the flow of blood per unit mass)[1,2]. Calibrated images were aligned with respective MRI CBF maps using a manual perspective transform. Whole brain, striatum and cortex ROIs were drawn on the MRI maps and the same ROIs were transferred to the autoradiography images for comparison.

References

1. Reivich M, Jehle J, Sokoloff L, et al. Measurement of regional cerebral blood flow with antipyrine-14C in awake cats. *J Appl Physiol* 1969; 27: 296–300.

2. Sakurada O, Kennedy C, Jehle J, et al. Measurement of local cerebral blood flow with iodo [14C] antipyrine. *Am J Physiol* 1978; 234: H59–H66.
