# Supplementary material for: Quantitative blood flow measurement in rat brain with multiphase arterial spin labelling magnetic resonance imaging
Source: J Cereb Blood Flow Metab. 2018 Mar 2;39(8):1557–69. doi: 10.1177/0271678X18756218 (PMC6681434; doi:10.1177/0271678X18756218)
Supplement: Supplementary figure -Supplemental material for Quantitative blood flow measurement in rat brain with multiphase arterial spin labelling magnetic resonance imaging [file Supplementary_figure.pdf]

Figure SI1:

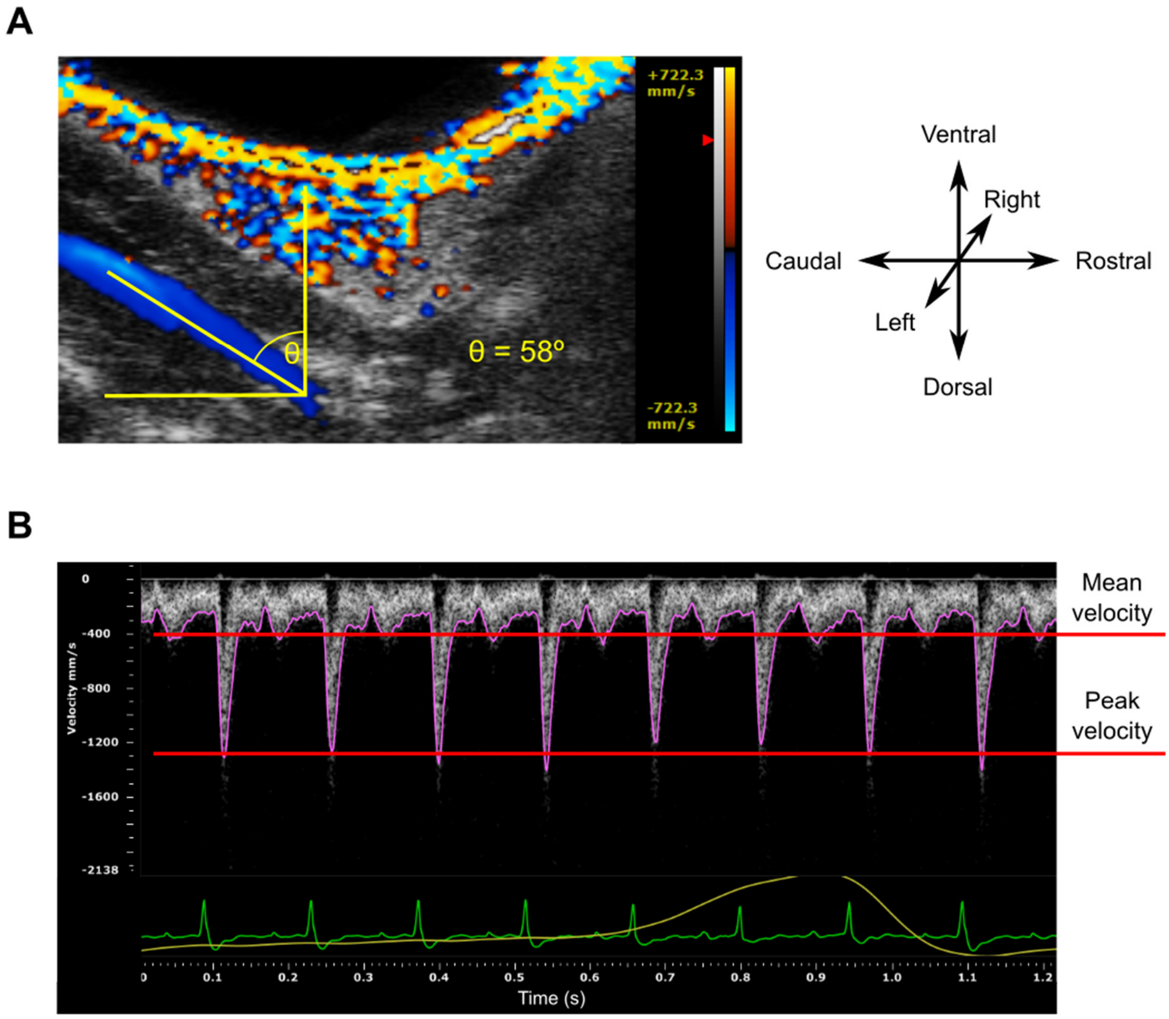

(A) Example ultrasound image of a rat neck with the carotid artery in blue indicating blood flowing away from the probe (top left to bottom right in this image). (B) Typical trace of blood flow velocity against time in an SD rat with mean and peak velocities indicated.

Figure SI2:

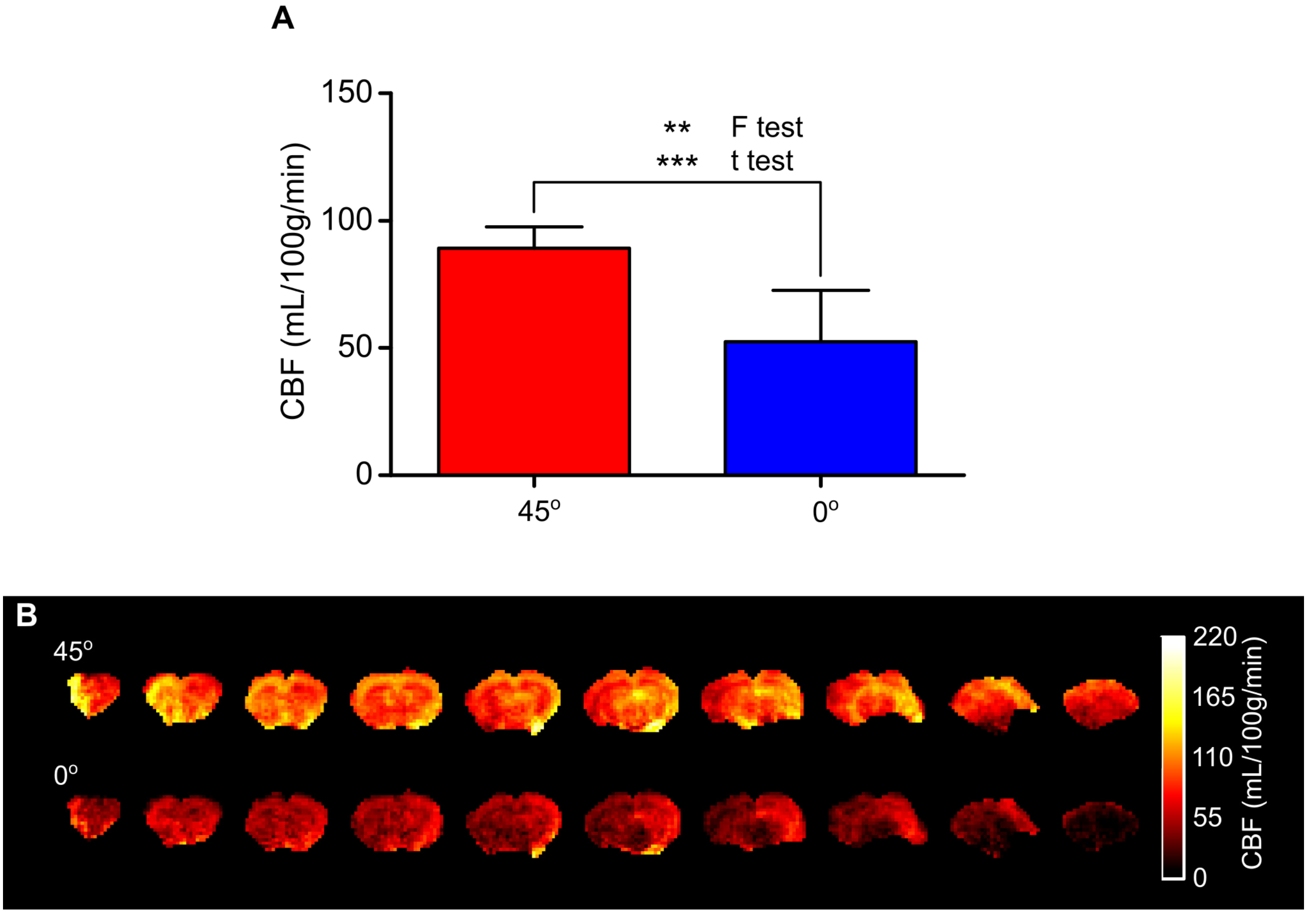

(A) CBF quantitation with a labelling plane angle of 0° or 45° with respect to the longitudinal axis of the animal; CBF values: t test ***p<0.001; variance: F test **p<0.01. (B) Example CBF map acquired with labelling angles of 45° and 0° from a female BDIX rat. All other acquisition parameters were identical. Four averages were acquired per image.

Figure SI3:

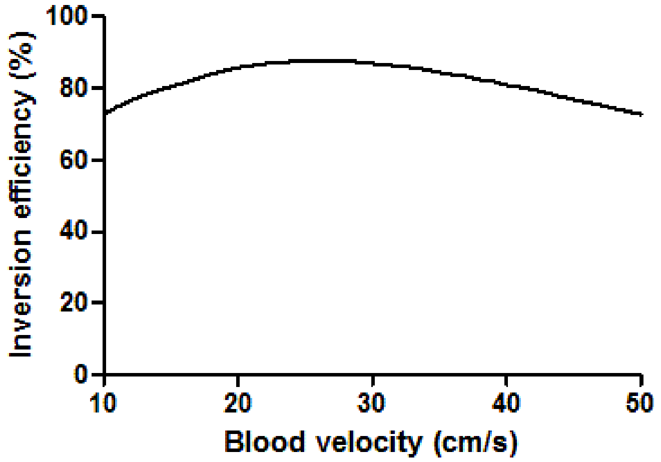

Predicted inversion efficiencies at a range of blood flow velocities with a 6.2 mm thick labelling plane and a flip angle of 40°. Other parameters as described in methods section.

Figure SI4:

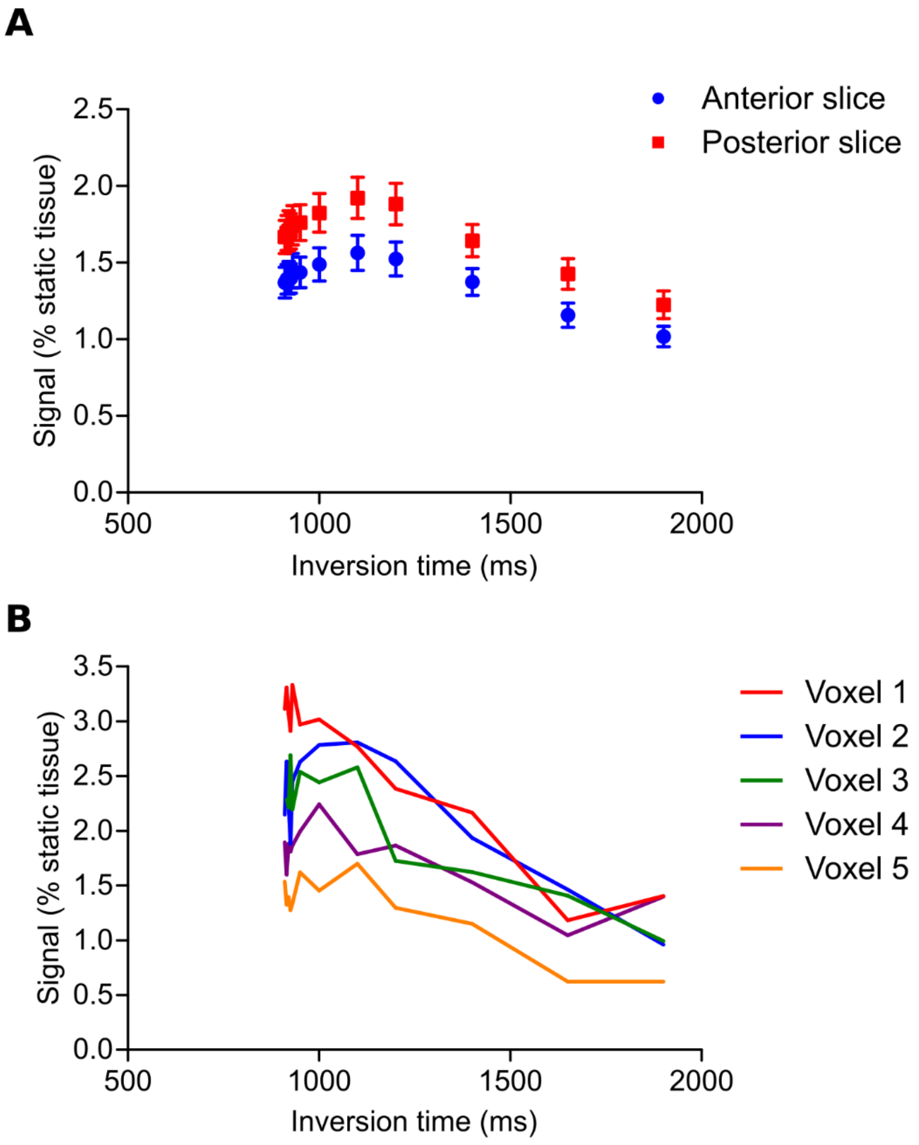

(A) Raw ASL signal as a percentage of static tissue for all animals at multiple inversion times (mean ± SEM) (B) Example data from individual voxels from five animals

Figure SI5:

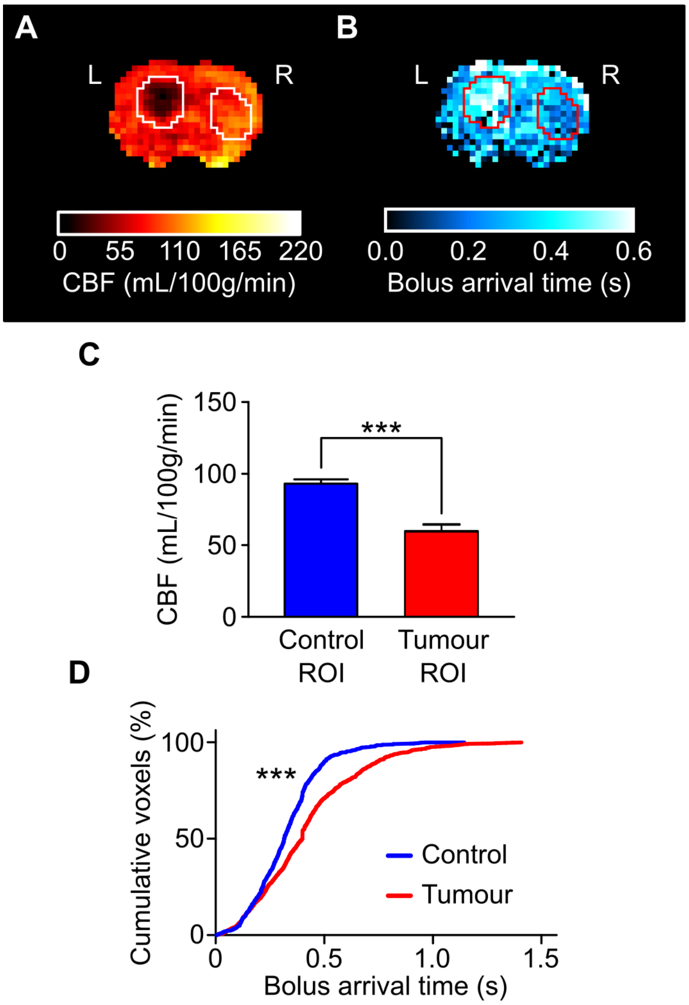

(A) Example CBF map in a tumour-bearing animal with tumour and contralateral ROIs indicated (B) Example bolus arrival time map in the same tumour-bearing animal with the same ROIs indicated (C) Group comparison between control (contralateral) and tumour ROIs (n=3, *** = p<0.001) (D) Cumulative frequency distributions for arrival times for voxels in tumour and control (contralateral) ROIs (*** = p<0.001)

Figure SI6:

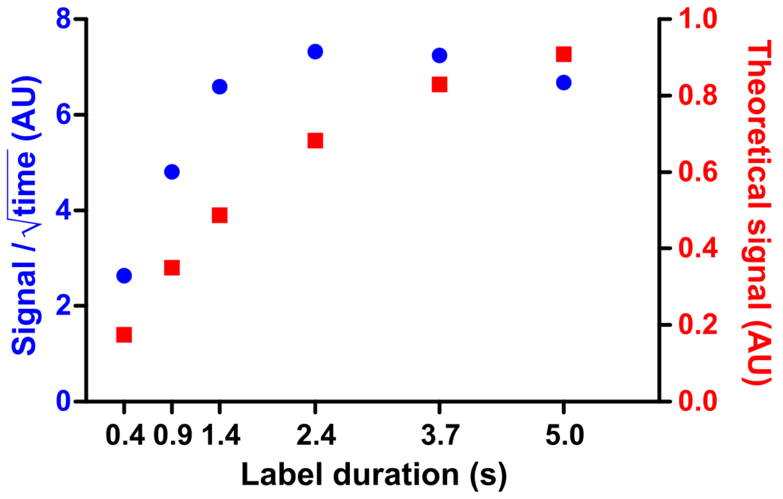

Accumulation of signal (AU) per unit time (i.e. efficiency of imaging) as a function of label duration (blue circles, left axis). Theoretical signal possible with increasing label durations (red squares, right axis).

Figure SI7:

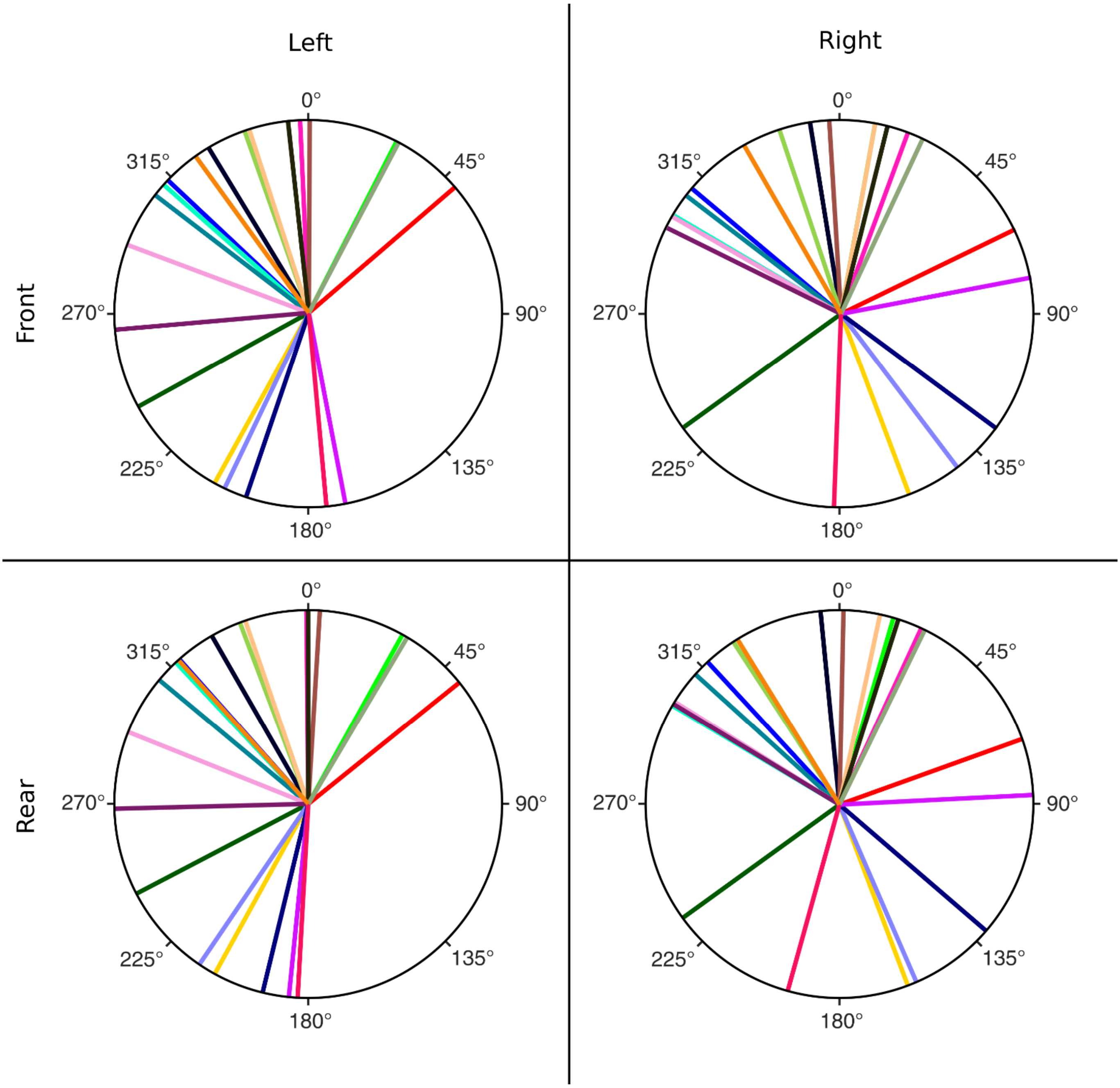

Observed optimal phase offsets for each of the four predominant supervoxel regions within rat brains. Each colour is unique and represents one rat within the study.
